# FBN1 Variant of Uncertain Significance in a Patient With a Marfan Spectrum Phenotype: A Case Report

**DOI:** 10.7759/cureus.115500

**Published:** 2026-08-31

**Authors:** Adriana I Ramirez Gandia, Emilio A Cepeda Terrasa, Natalio Izquierdo

**Affiliations:** 1 Department of Ophthalmology, Ponce Health Sciences University, Ponce, PRI; 2 Department of Ophthalmology, School of Medicine, Medical Sciences Campus, University of Puerto Rico, San Juan, PRI; 3 Department of Surgery, School of Medicine, Medical Sciences Campus, University of Puerto Rico, San Juan, PRI

**Keywords:** connective tissue disorder, ectopia lentis, fbn1, glaucoma suspect, marfan syndrome, variant of uncertain significance

## Abstract

Marfan syndrome (MFS) is an autosomal dominant multisystemic connective tissue disorder caused by pathogenic variants in the *FBN1 *gene and characterized by cardiovascular, ocular, and musculoskeletal manifestations. Ocular findings commonly include strabismus, refractive error, ectopia lentis, glaucoma, and retinal detachment. We report the case of a 14-year-old male patient with a variant of uncertain significance (VUS) in the *FBN1 *gene and a phenotype compatible with the Marfan spectrum. Clinical data were obtained from ophthalmic examination, ocular imaging, echocardiographic findings, and next-generation sequencing. The patient underwent ophthalmic evaluation as part of the workup for suspected MFS and had a best-corrected visual acuity of 20/30-1 and 20/30+1 in the right and left eyes, respectively. Slit-lamp examination showed minimal bilateral inferior lens subluxation (ectopia lentis) upon downgaze, and fundus examination showed bilateral optic disc cupping with otherwise normal posterior segment findings. Optical coherence tomography showed symmetric retinal nerve fiber layer thickness and a mild inter-eye difference in the cup-to-disc ratio. Based on bilateral optic disc cupping, the patient was considered a glaucoma suspect; however, glaucoma was not established. Systemic findings resulted in a Ghent systemic score of 7, while cardiovascular evaluation showed no evidence of aortic root dilation. Next-generation sequencing using an aortopathy panel identified a heterozygous *FBN1* c.7004G>A (p.Arg2335Gln) variant classified as a VUS. This case highlights the diagnostic challenges of interpreting *FBN1* variants of uncertain significance in patients with a highly suggestive Marfan spectrum phenotype and underscores the importance of comprehensive phenotypic assessment for clinical diagnosis and long-term management. The identified VUS cannot establish disease causality or provide definitive molecular confirmation. Rather, this case contributes phenotypic information regarding an unresolved *FBN1 *variant that may inform future genotype-phenotype correlation and variant re-evaluation as additional evidence becomes available.

## Introduction

Marfan syndrome (MFS) is an autosomal dominant multisystem connective tissue disorder caused by pathogenic variants in the *FBN1 *gene, primarily affecting the cardiovascular, ocular, and musculoskeletal systems [1-3]. Major cardiovascular manifestations include aortic root dilation predisposing to aortic dissection and rupture, mitral valve prolapse with or without regurgitation, tricuspid valve prolapse, mitral annular disjunction, and left ventricular dysfunction [4]. Ocular manifestations commonly include refractive errors, increased axial length, strabismus, cornea plana, glaucoma, ectopia lentis, and retinal detachment [2,3,5]. Musculoskeletal manifestations include disproportionately long extremities, arachnodactyly, dolichostenomelia, positive wrist and thumb signs, scoliosis, pectus excavatum or carinatum, and pes planus [3,6]. 

The *FBN1 *gene encodes fibrillin-1, a major structural glycoprotein of extracellular microfibrils that provides mechanical support to connective tissues throughout the body [2,7]. Fibrillin-1 is widely expressed in structures commonly affected in MFS, including the aortic wall, coronary arteries, skin fibroblasts, and ciliary zonules of the eye [6]. In addition to its structural role, fibrillin-1 regulates the bioavailability of transforming growth factor-beta (TGF-β) by sequestering latent TGF-β complexes within the extracellular matrix [2,7]. Pathogenic variants in *FBN1 *disrupt both connective tissue integrity and TGF-β regulatory function, resulting in abnormal connective tissue architecture and excessive TGF-β signaling that contribute to the multisystem manifestations characteristic of the syndrome [7,8]. 

MFS affects approximately 1 in 5,000 to 15,000 individuals worldwide and exhibits considerable phenotypic variability, which may complicate diagnosis and management [2]. The diagnosis is established using the revised Ghent criteria, which integrate clinical findings, family history, and molecular genetic testing [2,9]. Although more than 3,000 *FBN1 *variants have been reported, many remain incompletely characterized, and interpretation of variants of uncertain significance continues to present diagnostic challenges [2].

We present the case of a 14-year-old male patient with clinical features suggestive of a Marfan spectrum phenotype in whom genetic testing identified an *FBN1 *c.7004G>A variant classified as a variant of uncertain significance (VUS). Because a VUS cannot independently establish disease causality or provide definitive molecular confirmation, interpretation requires careful correlation with the patient's clinical phenotype and established diagnostic criteria. 

## Case presentation

A 14-year-old male patient was referred by his cardiologist to our clinic for ophthalmic evaluation as part of the workup for a phenotype compatible with MFS. Prior to referral, systemic features suggestive of a Marfan phenotype had been identified by his primary care physician, prompting evaluation by cardiology. As part of the subsequent cardiovascular assessment, the cardiologist ordered genetic testing using an aortopathy panel. The patient was then referred for ophthalmic evaluation to assess for ocular manifestations associated with MFS. Cardiovascular evaluation included a 12-lead electrocardiogram, which showed sinus bradycardia consistent with physiologic athletic conditioning as reported by the treating cardiologist (Figure 1). 

**Figure 1:**
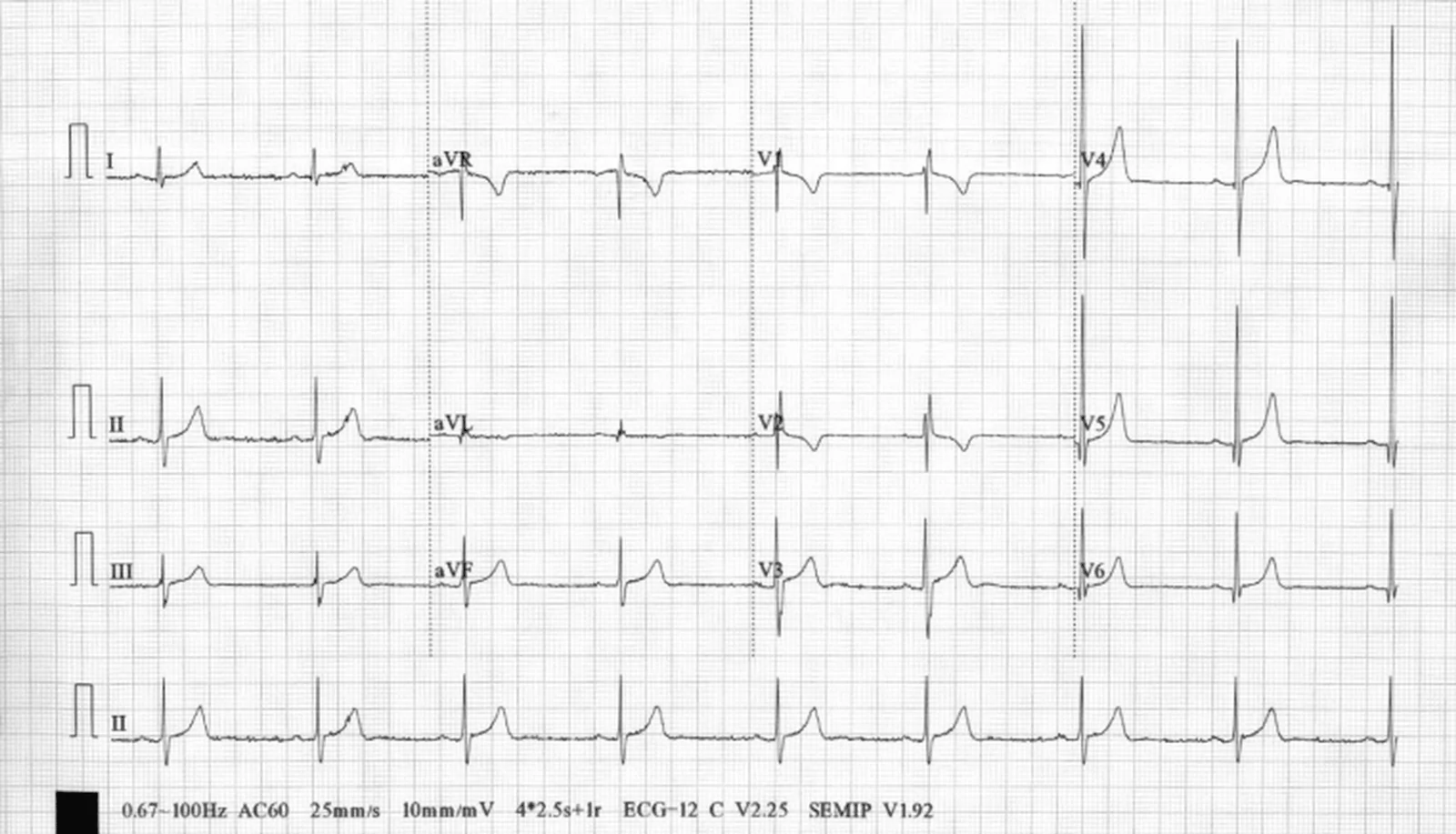
Twelve-lead electrocardiogram showing sinus bradycardia

Transthoracic echocardiography showed no atrial or ventricular septal defects, mitral valve prolapse, tricuspid valve prolapse, or aortic insufficiency. M-mode and Doppler echocardiographic measurements are summarized in Tables 1, 2 and are reported as documented in the clinical study. The aortic root measured 2.8 cm, with a reported Z-score of +0.53, indicating no evidence of aortic root dilation.

**Table 1:** Transthoracic echocardiographic M-mode measurements

Parameter	Value
Cardiac Dimensions	
Interventricular septal thickness in diastole (IVSd)	0.6 cm
Left ventricular internal diameter in diastole (LVIDd)	3.9 cm
Left ventricular posterior wall thickness in diastole (LVPWd)	0.7 cm
Interventricular septal thickness in systole (IVSs)	0.8 cm
Left ventricular internal diameter in systole (LVIDs)	2.8 cm
Left ventricular posterior wall thickness in systole (LVPWs)	1.0 cm
Relative wall thickness (RWT)	0.38
Left Ventricular Systolic Function	
End-diastolic volume (Teichholz)	66 mL
End-systolic volume (Teichholz)	30 mL
Stroke volume (Teichholz)	36 mL
Fractional shortening (%FS)	28%
Left ventricular ejection fraction (Teichholz)	55%
Aortic and Atrial Measurements	
Left atrial diameter	2.6 cm
Left atrium/Aorta ratio	0.93
Aortic root diameter	2.8 cm
Aortic arch diameter	2.3 cm

**Table 2:** Transthoracic echocardiographic Doppler measurements

Parameter	Value
Mitral Inflow Doppler	
Mitral valve E velocity	0.86 m/s
Mitral valve A velocity	0.18 m/s
Mitral E/A ratio	4.70
Tissue Doppler Imaging	
Septal E′ velocity	0.10 m/s
Lateral E′ velocity	0.11 m/s
Mean E′ velocity	0.07 m/s
E/E′ ratio	12.75
Left Ventricular Outflow Tract Doppler	
Aortic valve Vmax	1.18 m/s
Aortic valve peak gradient	5.55 mmHg
Descending aortic Vmax	1.13 m/s
Descending aortic peak gradient	5.13 mmHg
Pulmonary valve Vmax	0.80 m/s
Pulmonary valve peak gradient	2.55 mmHg
Tricuspid Inflow Doppler	
Tricuspid valve E velocity	0.54 m/s
Tricuspid valve A velocity	0.23 m/s
Tricuspid E/A ratio	2.38

Upon systemic examination, the patient had malar hypoplasia, a high-arched (gothic) palate, scoliosis, positive wrist and thumb signs, pes planus, and skin striae. When assessed according to the revised Ghent systemic scoring system, the presence of both wrist and thumb signs contributed three points, while scoliosis, pes planus, skin striae, and myopia greater than three diopters each contributed one point, resulting in a total systemic score of 7. Malar hypoplasia and the high-arched palate were documented as additional phenotypic features but did not independently contribute to the calculated systemic score. 

Upon initial comprehensive ophthalmic evaluation by one of the authors (NJI), the patient had best-corrected visual acuity of 20/30-1 and 20/30+1 in the right and left eyes, respectively. Cycloplegic refraction measured -3.75 -1.50 x 120° in the right eye and -2.50 -1.50 x 60° in the left eye. Intraocular pressure was 8 mmHg in both eyes. Extraocular motility was full, and confrontation visual fields were normal bilaterally. Upon slit-lamp examination, the patient had clear corneas, deep and quiet anterior chambers, irides without crypts, and minimal bilateral inferior lens subluxation (ectopia lentis), evident on downgaze. Fundus examination by indirect ophthalmoscopy showed bilateral optic disc cupping with normal retinal vasculature, macula, and peripheral retina. 

As shown in Figure 2, optic nerve optical coherence tomography showed an average retinal nerve fiber layer thickness of 90 µm in the right eye and 87 µm in the left eye. Average cup-to-disc ratios were 0.51 and 0.46 in the right and left eyes, respectively, and vertical cup-to-disc ratios were 0.45 and 0.37 in the right and left eyes, respectively. 

**Figure 2:**
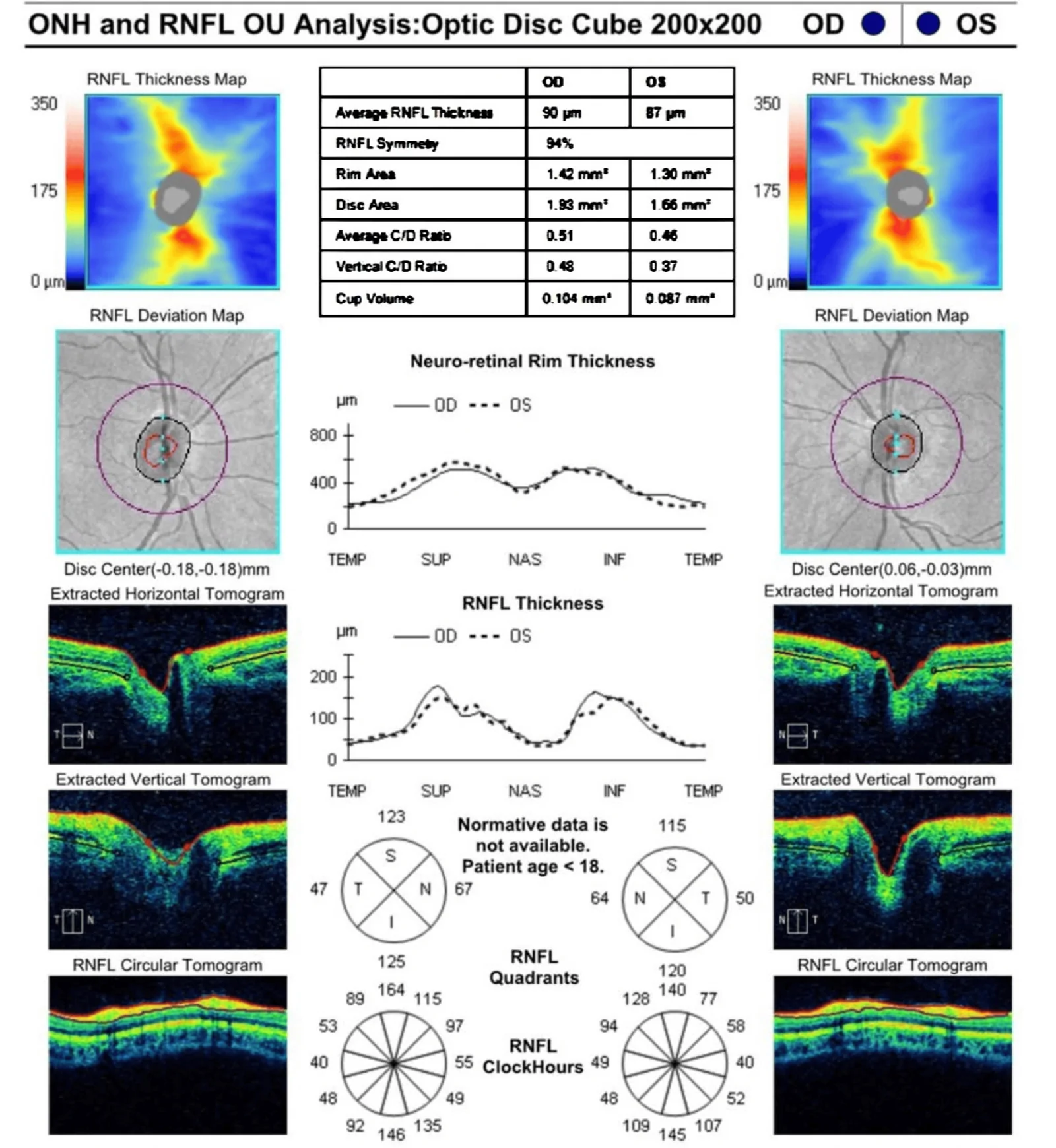
Optic nerve optical coherence tomography showing bilateral retinal nerve fiber layer thickness and optic nerve head analysis OCT: Optical coherence tomography; ONH: Optic nerve head; RNFL: Retinal nerve fiber layer

Next-generation sequencing using an Invitae aortopathy panel (Invitae Corporation, San Francisco, California, USA) identified a heterozygous *FBN1 *missense variant, NM_000138.5:c.7004G>A, resulting in an arginine-to-glutamine substitution at amino acid position 2335 (p.Arg2335Gln). The variant is located at 15q21.1 and is absent from the gnomAD exome dataset. The variant was classified by the testing laboratory as a VUS. Written informed consent for publication of this case and associated clinical information was obtained from the patient's legal guardians. 

## Discussion

MFS was first described by French pediatrician Antoine-Bernard Marfan in 1896 and is now recognized as a multisystem connective tissue disorder involving the musculoskeletal, cardiovascular, and ocular systems [1,10]. The syndrome is characterized by variable manifestations, including skeletal overgrowth, aortic root dilation, and ocular findings [1].

The revised Ghent nosology places particular emphasis on cardiovascular, ocular, genetic, and systemic manifestations when establishing the diagnosis of MFS [9]. In our patient, findings including positive wrist and thumb signs, scoliosis, pes planus, skin striae, and myopia greater than three diopters resulted in a systemic Ghent score of seven points, meeting the threshold for systemic involvement [9]. However, a systemic score of ≥7 represents systemic involvement and does not independently establish a diagnosis of MFS. Diagnostic interpretation must therefore integrate the systemic score with the patient's cardiovascular, ocular, genetic, and family history findings according to the revised Ghent criteria [9].

Since cardiovascular involvement remains the principal determinant of diagnosis in sporadic cases, cardiovascular assessment plays a central role in the evaluation of suspected MFS [9]. In our patient, cardiovascular evaluation showed normal cardiac anatomy and function, with no evidence of aortic root dilation (aortic root Z-score of +0.53), valvular abnormalities, or septal defects at the time of assessment. Although these findings do not currently provide cardiovascular support for the diagnosis, the revised Ghent nosology recognizes the age-dependent nature of Marfan manifestations, with cardiovascular manifestations potentially evolving with age, particularly in pediatric patients [2,9]. Therefore, the absence of aortic root dilation at the present evaluation should be interpreted in the context of the patient's age and does not preclude its development over time. Continued echocardiographic surveillance is warranted to monitor for evolving cardiovascular involvement [2,9].

Ocular manifestations in patients with MFS include refractive errors, strabismus, ectopia lentis, increased axial length, glaucoma, cornea plana, and retinal detachment [5,11,12]. Ectopia lentis is one of the hallmark ocular manifestations and represents a major diagnostic criterion within the revised Ghent nosology [2,9,11]. Tinkle and co-workers reported that ectopia lentis is often a presenting feature of MFS and is very uncommon in the general population [2]. Our patient had minimal bilateral inferior lens subluxation (ectopia lentis) and bilateral optic disc cupping suspicious for glaucoma. These findings are consistent with previously reported ocular manifestations in patients with MFS and support the presence of a Marfan spectrum phenotype. 

Studies evaluating patients with MFS have shown marked phenotypic and genetic variability, emphasizing the importance of careful clinical correlation when interpreting molecular findings [1,9]. In our patient, next-generation sequencing using an aortopathy panel identified an *FBN1 *c.7004G>A (p.Arg2335Gln) variant classified as a VUS. Interpretation of variants of uncertain significance remains one of the major challenges in clinical genetics, particularly in disorders exhibiting marked phenotypic variability such as MFS [13,14]. Although pathogenic and likely pathogenic *FBN1 *variants may provide molecular support for the diagnosis of MFS when interpreted within the appropriate clinical context, a VUS cannot be used as definitive molecular confirmation of the disease [13,14].

According to the revised Ghent nosology, the combination of bilateral ectopia lentis and systemic involvement alone is insufficient to establish a diagnosis of MFS in the absence of aortic root dilation or an *FBN1 *pathogenic variant previously associated with aortic disease [9]. Nevertheless, our patient showed bilateral ectopia lentis together with a systemic Ghent score of seven points, supporting a Marfan spectrum phenotype despite the current classification of the identified *FBN1 *variant as a VUS [9]. These phenotypic findings may become increasingly valuable for future variant interpretation as additional genetic and clinical evidence becomes available [13,14].

Previous studies have established the biological association between pathogenic *FBN1 *variants and ectopia lentis [15]. In our patient, the ectopia lentis was minimal and evident on downgaze, compatible with the early ocular manifestations described in pediatric patients with MFS [2]. The *FBN1 *gene encodes fibrillin-1, a major extracellular matrix glycoprotein involved in connective tissue integrity and regulation of transforming growth factor-beta [1,7]. Fibrillin-1 is highly expressed in the ciliary zonules of the eye, which are responsible for lens suspension [7,15]. Disruption of fibrillin-1 structure or function may weaken zonular integrity and predispose to lens subluxation, providing a biological basis for the association between pathogenic *FBN1 *variants and ectopia lentis [7]. 

Studies by Maumenee and co-workers reported glaucoma in patients with MFS. Our patient had bilateral optic nerve cupping and was therefore considered a glaucoma suspect; however, glaucoma was not established at the time of evaluation. Although glaucoma is a recognized ocular manifestation of MFS, the mechanisms underlying its development remain incompletely understood [16,17]. Fibrillin-1 is expressed not only in the ciliary zonules of the eye but also within the trabecular meshwork and other components of the aqueous humor outflow pathway [7,16]. Alterations in fibrillin-1 have been proposed to contribute to glaucoma susceptibility by affecting aqueous humor outflow or microfibril-related signaling pathways such as TGF-β regulation [7,16]. Longitudinal follow-up with visual field testing and serial optic nerve imaging will be important to determine whether progressive changes consistent with glaucoma develop over time.

The *FBN1 *c.7004G>A (p.Arg2335Gln) variant is a missense variant resulting in the substitution of arginine by glutamine at amino acid position 2335 of fibrillin-1. The variant is absent from the gnomAD exome dataset and remains classified as a VUS by multiple ClinVar submitters [18]. Available in silico analysis supports a potentially deleterious effect on protein structure or function [18]. However, the substitution does not affect a cysteine residue within a calcium-binding epidermal growth factor-like domain, where cysteine substitutions represent a common mechanism among pathogenic *FBN1 *missense variants [18,19]. Available ClinVar submissions have evaluated the variant in the context of MFS and related *FBN1-*associated aortopathy phenotypes, but the currently available clinical and molecular evidence remains insufficient to establish pathogenicity [18]. Segregation analysis, functional characterization, reports of unrelated affected individuals harboring the same variant, and periodic variant re-evaluation may provide additional evidence relevant to its future classification [13,14]. Additionally, long-term cardiovascular follow-up may further clarify the patient’s evolving clinical phenotype. Therefore, documentation of genotype-phenotype correlations in patients harboring rare or uncertain variants is essential and may contribute to future variant interpretation efforts. 

This case highlights the diagnostic challenges associated with interpreting variants of uncertain significance in the setting of a highly suggestive clinical phenotype. Although the *FBN1 *variant has been previously submitted to ClinVar, it remains classified as a VUS, and the available evidence is insufficient to establish a definitive genotype-phenotype relationship [18]. The value of this report therefore lies not in establishing the pathogenicity of the variant, but in documenting its occurrence in a patient with bilateral ectopia lentis and a systemic Ghent score of 7. This phenotypic characterization adds clinical information that may contribute to future genotype-phenotype correlation and variant re-evaluation as additional cases and molecular evidence become available. Additionally, this case underscores the importance of comprehensive ophthalmic and systemic evaluation in patients with suspected heritable connective tissue disorders [3].

Patients with MFS or Marfan spectrum phenotypes benefit from multidisciplinary care involving primary care, cardiology, ophthalmology, and genetics as part of ongoing surveillance and risk reduction [2,9]. This coordinated approach is particularly important in pediatric patients, in whom cardiovascular and ocular manifestations may evolve over time [2,9]. 

This report is limited by its description of a single patient, which restricts the generalizability of the findings. Additionally, parental testing and family segregation analysis were not performed. Therefore, it remains unknown whether the *FBN1* variant was inherited or occurred de novo. The absence of segregation data limits assessment of the relationship between the variant and the observed phenotype and represents an important limitation in its interpretation. Because the identified *FBN1 *variant remains classified as a VUS, it cannot currently provide definitive molecular confirmation of MFS [13,14]. Nevertheless, the presence of bilateral ectopia lentis together with a systemic Ghent score of seven points supports a clinical Marfan spectrum phenotype despite the absence of definitive molecular confirmation. The identified *FBN1 *variant should therefore be regarded as unresolved molecular information rather than as an explanation for the patient's phenotype. 

Future studies involving larger patient cohorts, long-term follow-up, continued reporting of patients harboring uncertain *FBN1 *variants, and detailed phenotypic characterization are essential for improving genotype-phenotype correlations. Such evidence may facilitate future variant reclassification and ultimately improve the molecular diagnosis and clinical management of patients with suspected MFS.

## Conclusions

This case describes a patient with musculoskeletal features and bilateral ectopia lentis, consistent with a Marfan spectrum phenotype, as well as optic nerve cupping, who harbors an *FBN1 *c.7004G>A variant classified as a variant of uncertain significance. Although the identified variant cannot currently provide definitive molecular confirmation of MFS, the patient’s ocular and systemic findings highlight the importance of comprehensive phenotypic assessment when interpreting uncertain genetic results. Continued cardiovascular, ophthalmic, and genetic surveillance is essential, particularly in pediatric patients whose manifestations may evolve over time. Furthermore, documentation of patients harboring rare or uncertain *FBN1 *variants may contribute to future genotype-phenotype correlation and facilitate variant re-evaluation as additional evidence becomes available. 
